# Soluble TREM2 Concentrations in the Cerebrospinal Fluid Correlate with the Severity of Neurofibrillary Degeneration, Cognitive Impairment, and Inflammasome Activation in Alzheimer’s Disease

**DOI:** 10.3390/neurolint15030053

**Published:** 2023-07-07

**Authors:** Ena Španić Popovački, Mirjana Babić Leko, Lea Langer Horvat, Klara Brgić, Željka Vogrinc, Marina Boban, Nataša Klepac, Fran Borovečki, Goran Šimić

**Affiliations:** 1 Department of Neuroscience, Croatian Institute for Brain Research, University of Zagreb School of Medicine, Šalata 12, 10000 Zagreb, Croatia; 2 Department of Neurosurgery, University Hospital Centre Zagreb, Kišpatićeva 12, 10000 Zagreb, Croatia; 3 Laboratory for Neurobiochemistry, Department of Laboratory Diagnostics, University Hospital Centre Zagreb, Kišpatićeva 12, 10000 Zagreb, Croatia; 4 Department of Neurology, University Hospital Centre Zagreb, Kišpatićeva 12, 10000 Zagreb, Croatia; 5 School of Medicine, University of Zagreb, 10000 Zagreb, Croatia

**Keywords:** inflammasome, microglia, mild cognitive impairment, plasma samples, tau protein, ELISA method

## Abstract

Background: Individuals with specific *TREM2* gene variants that encode for a Triggering Receptor Expressed on Myeloid cells 2 have a higher prevalence of Alzheimer’s disease (AD). By interacting with amyloid and apolipoproteins, the TREM2 receptor regulates the number of myeloid cells, phagocytosis, and the inflammatory response. Higher *TREM2* expression has been suggested to protect against AD. However, it is extremely difficult to comprehend TREM2 signaling in the context of AD. Previous results are variable and show distinct effects on diverse pathological changes in AD, differences between soluble and membrane isoform signaling, and inconsistency between animal models and humans. In addition, the relationship between TREM2 and inflammasome activation pathways is not yet entirely understood. Objective: This study aimed to determine the relationship between soluble TREM2 (sTREM2) levels in cerebrospinal fluid (CSF) and plasma samples and other indicators of AD pathology. Methods: Using the Enzyme-Linked Immunosorbent Assay (ELISA), we analyzed 98 samples of AD plasma, 35 samples of plasma from individuals with mild cognitive impairment (MCI), and 11 samples of plasma from healthy controls (HC), as well as 155 samples of AD CSF, 90 samples of MCI CSF, and 50 samples of HC CSF. Results: CSF sTREM2 levels were significantly correlated with neurofibrillary degeneration, cognitive decline, and inflammasome activity in AD patients. In contrast to plasma sTREM2, CSF sTREM2 levels in the AD group were higher than those in the MCI and HC groups. Moreover, concentrations of sTREM2 in CSF were substantially higher in the MCI group than in the HC group, indicating that CSF sTREM2 levels could be used not only to distinguish between HC and AD patients but also as a biomarker to detect earlier changes in the MCI stage. Conclusions: The results indicate CSF sTREM2 levels reliably predict neurofibrillary degeneration, cognitive decline, and inflammasome activation, and also have a high diagnostic potential for distinguishing diseased from healthy individuals. To add sTREM2 to the list of required AD biomarkers, future studies will need to include a larger number of patients and utilize a standardized methodology.

## 1. Introduction

Carriers of certain *TREM2* gene variants (R47H, D87N, L211P, H157Y, R62H, and T96K) have an increased risk of Alzheimer’s disease (AD). Therefore, the TREM2 receptor is commonly investigated in the context of AD [1,2]. After *APOE* gene variants [3], the R47H variant of *TREM2* is the second most common risk factor for AD, and it can increase the risk of developing AD by two- to fourfold [2]. ApoE is one of the TREM2 receptor ligands, and its interaction, if altered, can have a significant effect on the onset of AD symptoms [4]. The TREM2 receptor is indispensable for interacting with apolipoproteins and amyloid-β (Aβ), and regulating the number of myeloid cells, phagocytosis, and the inflammatory response [1,2]. TREM2 is also essential for synaptic pruning and regulates synaptic clearance via phagocytosis, whereas the absence of TREM2 results in aberrant synaptic clearance [4]. Microglial cholesterol metabolism also depends on TREM2 signaling, as TREM2 deficiency is associated with impaired intracellular cholesterol storage and efflux [5]. Intriguingly, TREM2 indirectly regulates the myelinating process by influencing cholesterol metabolism and clearing myelin debris [5]; this is severely disrupted by the *TREM2* p.Q33X mutation in microglia, which interferes with lysosomal function and contributes to Nasu–Hakola disease [6,7]. The downstream signaling of TREM2 induces transcriptional changes that promote the transition from a homeostatic to a disease-associated microglia phenotype (DAM) [8]. The TREM2 receptor also mediates autophagy, one of the mechanisms for degrading cellular proteins, particularly abnormally aggregated proteins, which is impaired in AD [9]. Humans with TREM2 risk variants and TREM2-deficient mice accumulate vesicles resembling autophagy [9].

The protective effect of increased *TREM2* expression against AD symptoms has previously been reported [10]. The TREM2 receptor interacts with the Aβ to initiate its phagocytosis [11]. Higher levels of cerebrospinal fluid (CSF) sTREM2 are associated with delayed Aβ accumulation in the brain, as measured by amyloid-PET scan [12]. In the 5XFAD model of AD, protracted TREM2 stimulation via injection of human TREM2 (hTREM2) agonistic mAb (AL002c) attenuated pathological changes in Aβ and neurite injury [13]. However, a recent study suggests that chronic TREM2 antibody administration does not influence Aβ plaque burden [14]. In addition, chronic TREM2 activation increased the dissemination of tau neurofibrillary degeneration and loss of the synapses surrounding plaques in the region with the highest pathological tau changes and neuritic dystrophy [14]. In contrast, the soluble form of the TREM2 receptor (sTREM2) promoted Aβ plaque degradation [15], indicating that two receptor isoforms may play distinct functions in relation to pathological changes and disease stages. In accordance with that statement, a recent study on APP/PS1 mice demonstrated that increased TREM2 activation indirectly inhibits tau protein phosphorylation and neuronal loss by inhibiting glycogen synthase kinase-3β, the major actor of tau hyperphosphorylation [16]. In addition, AD-associated microglial activation stage 2 markers in non-demented individuals are predictive of a delayed accumulation of tau [17]. The increased activity of TREM2 in the transgenic mouse model is associated with decreased expression of genes essential for the pro-inflammatory response of microglia [18] and can increase its phagocytotic activity [12,13]. *TREM2* expression and inflammasome activation produce contradictory outcomes [19,20,21,22,23]. Microglia cells with the R47H variant have a diminished rate of inflammasome activation [19], whereas macrophages with higher *TREM2* expression inhibit inflammasome activation [20]. In contrast, their pyroptosis increases in the absence of TREM2 [21]. A similar effect was observed in C57/BL6 mice, where overexpression of *TREM2* attenuated postoperative neuroinflammation [24]. *TREM2* overexpression also ameliorates LPS-induced oxidative stress and inflammation in the BV2 cell line [25]. In contrast, one study discovered that the AD mouse model *TREM2* knock-out (APPPS1; *Trem2*^−/−^) has decreased expression of the pro-inflammatory cytokines IL-1β and IL-6 and increased expression of anti-inflammatory markers associated with reduced Aβ and tau protein pathological alterations [22]. Microglia activated by high glucose levels increase *TREM2* expression and pro-inflammatory cytokine release, whereas downregulation of *TREM2* reduces inflammation caused by high glucose levels [23]. Soluble TREM2 (sTREM2) is a form of TREM2 receptor that is released after its extracellular domain is cleaved by ADAM10 and ADAM17 metalloproteases [11], and its function remains poorly understood. Another process can be involved in generating sTREM2, namely, the soluble form of the receptor can be produced by transcription of the alternative transcript lacking a transmembrane domain [26], and it appears that approximately 25% of the sTREM2 may be due to the expression of this isoform. As discussed, TREM2 shedding from the membrane may function as a negative regulator of TREM2 signaling [27]. It is still debatable whether the soluble form of TREM2 has an agonistic effect or whether it competes with the presumably protective full-length membrane TREM2 [11,27]. Additionally, it is not known if sTREM2 CSF levels are positively correlated with CSF Apoptosis-associated Speck-like protein Containing a CARD (CAspase Recruitment Domain) (ASC) protein levels, which would indicate an association between the release of the sTREM2 form and the activation of the inflammasomes. ASC is an adaptor protein that plays a crucial role in the assembly of the inflammasome, a multiprotein complex involved in the activation of inflammatory responses, regulation of the production of pro-inflammatory cytokines, and the initiation of immune responses [19,20,21]. Therefore, revealing the correlation of CSF sTREM2 with CSF ASC protein levels was also one of the objectives of this study.

It has been hypothesized that sTREM2 initiates the survival of microglia and the secretion of pro-inflammatory cytokines [28]. In the 5xFAD transgenic mouse model, injection of sTREM2 into the hippocampus increases microglial clustering around amyloid plaques and reduces amyloid plaque load by uptake and degradation of Aβ, thus rescuing deficits of spatial memory and long-term potentiation [15]. However, the depletion of microglia abolishes the neuroprotective effect of sTREM2 [15]. Recently, a rare p.H157Y variant of *TREM2* that gives rise to cleavage sites at the extracellular domain was identified; this variant is associated with increased production of sTREM2 and an increased risk of AD [29]. In vivo experiments with *Trem2* H157Y knock-in mice revealed that increased levels of sTREM2 are advantageous for the animals, resulting in improved synapse function and amelioration of amyloid pathological alterations [29]. However, the study did not include other pathological factors, such as tau protein changes, which could be one explanation for why this mutation has a different effect in humans [29]. In a separate study examining the effect of the R47H variant on tau pathology in the PS19 mouse model of tauopathy expressing either human common TREM2 or the R47H variant, it was discovered that impaired TREM2 signaling (in the case of the R47H variant) significantly attenuated neurodegenerative changes caused by tau pathology. It is hypothesized that altered TREM2 receptor signaling influences microglial activation and, as a result, ameliorates microglia-mediated degeneration [30]. It appears that tau neurofibrillary degeneration associated with TREM2 signaling is highly dependent on ApoE variants [31]. TREM2 knock-out P301S tau mice with an expressed ApoE4 variant have significantly higher neurodegenerative and tau protein alterations than mice with the same ApoE variant and an expressed *TREM2* gene [31]. In the THY-Tau22 transgenic murine model of tauopathy, TREM2 deficiency decreased microglial activation, which exacerbated pathological changes in later stages [32]. The TREM2 signaling pathways are extraordinarily complex, as evidenced by their varying effects on diverse pathological alterations, differences in signaling between soluble and membrane isoforms signaling, and discordance between animal models and human AD patients. In amyloid mouse models of AD, for instance, brain expression or injection of wild-type sTREM2 decreases amyloid pathological changes, indicating that wild-type sTREM2 is protective against amyloid pathology [33].

To determine whether sTREM2 protects against AD and at what stage of the disease, it is crucial to conduct additional longitudinal studies on the AD population. Analysis of the TREM2 levels in the CSF generally revealed elevated levels in AD patients; however, other studies did not confirm these findings [34], and some reported variable TREM2 levels dependent on the disease stage, with the highest levels typically associated with earlier symptomatic stages (for review, see [33]). Intriguingly, an increase in TREM2 CSF levels correlates positively with total and phosphorylated tau CSF levels, and inversely with Aβ levels [1,34,35,36,37]. Lower Aβ-PET signaling was associated with higher CSF TREM2 levels, indicating that microglial activity may be protective against the formation of Aβ plaques [12]. Therefore, sTREM2 may be a useful biomarker of the pathological state of AD; however, future research should include additional studies with a greater number of patients and more uniform analysis procedures. This study aimed to determine the relationship between sTREM2 levels in CSF and plasma samples and other markers of AD pathology and the activation of the inflammasomes.

## 2. Materials and Methods

### 2.1. Cerebrospinal Fluid and Blood Collection

This study included patients admitted to the University Hospital Center “Zagreb” in Zagreb, Croatia. AD diagnosis was based on NIA-AA (National Institute on Aging-Alzheimer’s Association guidelines for the neuropathologic assessment of Alzheimer’s disease) [38,39], NINCDS-ADRDA (National Institute of Neurological and Communicative Disorders and Stroke and Alzheimer’s Disease and Related Disorders Association), and DSM-IV-TR (Diagnostic and Statistical Manual of Mental Disorders) criteria [40]. The diagnosis of MCI (mild cognitive impairment) was determined by criteria described by Petersen et al. [41] and Albert et al. [42]. Samples were collected during a routine patient examination at one time point (a cross-sectional study). CSF samples were obtained by lumbar puncture (performed at intervertebral spaces L3/L4 or L4/L5). Following 10 min of centrifugation at 2000× *g*, CSF samples were aliquoted and stored at −80 °C in polypropylene tubes. Venous blood samples were collected in the morning on an empty stomach. Samples were collected using plastic syringes containing 1 mL of acid citrate dextrose as an anticoagulant. Thrombocyte-free plasma was extracted through centrifugation, first at 1100× *g* for 3 min and then at 5087× *g* for 15 min. Plasma samples were stored at −20 °C. All procedures were approved by the Ethical Committee of the Clinical Hospital Center “Zagreb” (protocol no. 02/21 AG, class 8.1–18/82-2 from 24 April 2018, and protocol no. 02/21 AG, class 8.1–19/201-2 from 23 September 2019) and the Central Ethical Committee (Institutional Review Board) of the University of Zagreb School of Medicine (protocol no. 380-59-10106-18-111/126, class 641-01/18-02/01 from 20 June 2018, and protocol no. 380-59-10106-19-111/251, class 641-01/19-02/01 from October 2019). We analyzed 98 AD, 35 MCI, and 11 healthy controls (HC) plasma samples, as well as 155 AD CSF samples, 90 MCI CSF samples, and 50 HC CSF samples.

### 2.2. ELISA Procedure

Levels of the biomarkers (sTREM2, ASC, p-tau_181_, t-tau, and Aβ_1-42_) in CSF and sTREM2 in plasma samples were determined with an Enzyme-Linked Immunosorbent Assay (ELISA) according to manufacturer protocols for:

- TREM2 (Human TREM2 ELISA Kit, Abcam, Cambridge, UK);
- Aβ_1-42_ (Innotest β-amyloid1-42, Fujirebio, Gent, Belgium);
- total tau (Innotest hTau Ag, Fujirebio, Gent, Belgium);
- p-tau_181_ (Innotest Phospho-Tau(181P), Fujirebio, Gent, Belgium);
- ASC (Human PYCARD/ASC/TMS1 Sandwich ELISA, LSBio, Seattle, WA, USA).

Aβ_1-42_, t-tau, p-tau_181_, and ASC ELISA measurements were performed on 96-well plates coated with capture antibodies (indirect capture method). Samples and standards were added and incubated in the plates. The ASC ELISA samples were diluted 1:2. After incubation, the detection antibody was added to the washed wells. For the indirect detection of Aβ_1-42_, t-tau, p-tau_181_, and ASC, biotin-labeled detection antibodies were used in conjunction with the streptavidin-HRP complex.

The sTREM2 ELISA kit consisted of a 96-well plate coated with an anti-tag antibody that immobilized the complex of capture antibody-analyte-detector antibody. After adding samples (1:50 dilution) and standards to the wells, a capture/detector antibody mixture was added. HRP chromogenic substrate was applied following incubation and washing. All analytes’ absorbances were measured at 450 nm at Glomax Explorer (Promega, Madison, WI, USA), and protein concentrations were calculated using a 4-parameter algorithm in GraphPad Prism 8.0 (GraphPad Software Inc., San Diego, CA, USA).

### 2.3. Statistical Analysis

GraphPad Prism 8.0 (GraphPad Software Inc., San Diego, CA, USA) software was used for statistical analyses, with the level of statistical significance set at α = 0.05. Because of the small number of subjects in some groups, non-parametric statistical tests were used. A non-parametric Kruskal–Wallis test with the Dunn–Bonferroni post-hoc test was used for the comparison of the sTREM2 levels between the groups in CSF and plasma samples. For the correlation analyses, we used a non-parametric Spearman correlation test, and the correlation of the parameters was tested in all subjects. The diagnostic sensitivity, specificity, and cut-off value for CSF sTREM2 were measured by the analysis of the Receiver Operating Characteristic (ROC) Area Under Curve (AUC). The best cut-off value was determined when the sum of sensitivity and specificity was maximized.

## 3. Results

In Table 1, the basic characteristics of the MCI, AD, and HC groups are presented.

### 3.1. sTREM2 Levels in CSF and Plasma Samples

**Figure 1:**
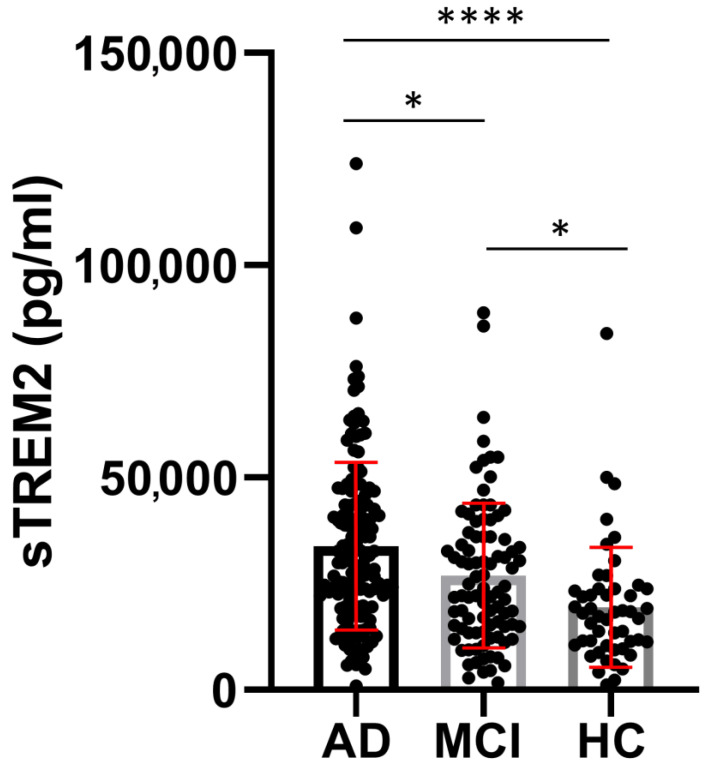
Significantly higher levels of CSF sTREM2 in AD compared to MCI (* *p* = 0.01), and HC (**** *p* < 0.0001), as well as in the MCI versus HC group (* *p* = 0.01). AD, Alzheimer’s disease group; CSF, cerebrospinal fluid; HC, healthy control groups, MCI, mild cognitive impairment group; sTREM2, soluble Triggering Receptor Expressed on Myeloid cells 2.

**Figure 2:**
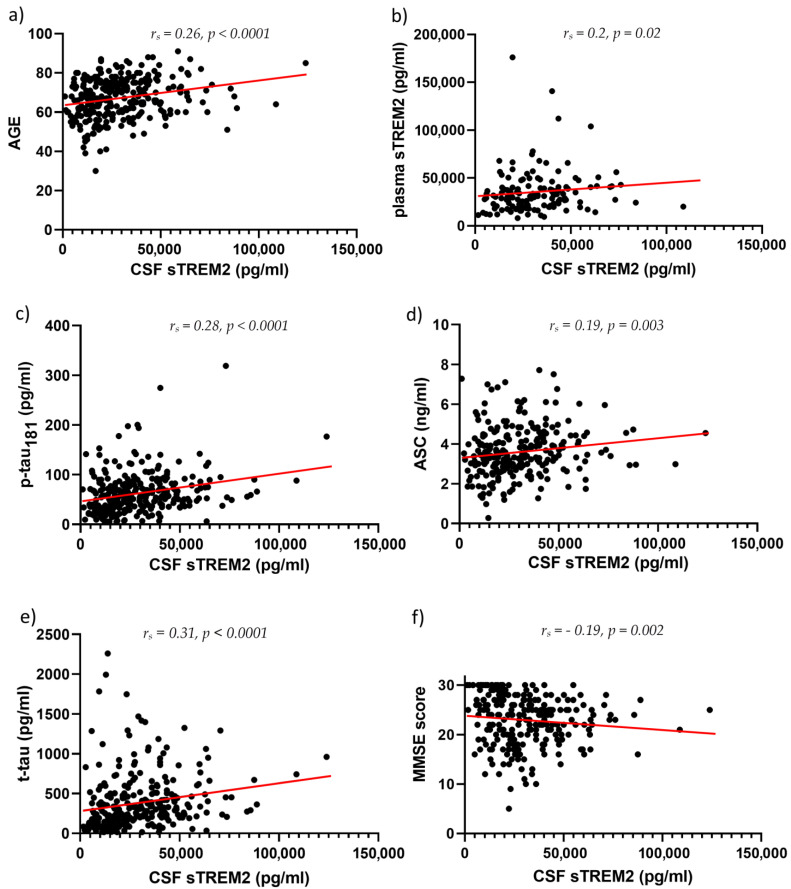
CSF sTREM2 levels correlate positively and highly significantly with age (r_s_ = 0.26, *p* < 0.0001) (**a**), positively and significantly with plasma sTREM2 levels (r_s_ = 0.2, *p* = 0.02) (**b**), positively and highly significantly with p-tau_181_ (r_s_ = 0.28, *p* < 0.0001) (**c**), positively and significantly with ASC levels (r_s_ = 0.19, *p* = 0.003) (**d**), positively and highly significantly with t-tau protein concentrations (r_s_ = 0.31, *p* < 0.0001) (**e**), and negatively and significantly with MMSE score (r_s_ = −0.19, *p* = 0.002) (**f**). ASC, Apoptosis-associated Speck-like protein Containing a CARD (CAspase Recruitment Domain); MMSE, Mini-Mental State Examination; p-tau, tau phosphorylated at epitope 181; sTREM2, soluble Triggering Receptor Expressed on Myeloid cells 2; t-tau, total tau proteins.

**Figure 3:**
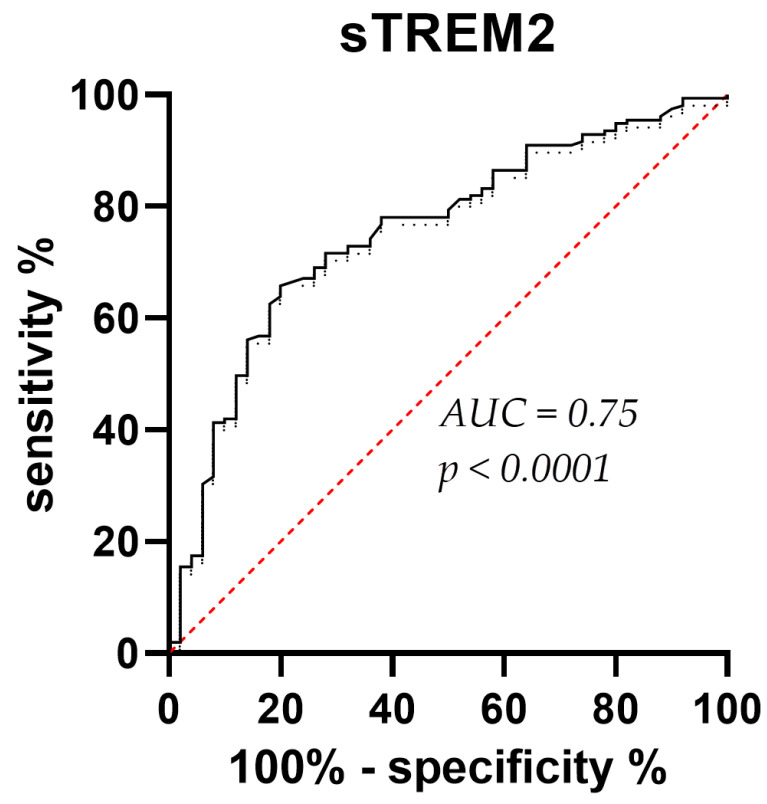
ROC curve for CSF sTREM2 levels. The sensitivity and specificity of sTREM2 in CSF as a biomarker for AD were 65.81% and 80.00%, respectively. The best cut-off value was determined when the sum of sensitivity and specificity was maximized. This cut-off value is 23,950 pg/mL. AUC = 0.75; *p* < 0.0001. AD, Alzheimer’s disease; AUC, Area Under Curve; ROC, Receiver Operating Characteristic; sTREM2, sTREM2, soluble Triggering Receptor Expressed on Myeloid cells 2.

CSF sTREM2 concentrations were significantly higher in AD than in MCI (*p* = 0.01) and HC (*p* < 0.0001), and in MCI than in the HC group (*p* = 0.01) (Figure 1). Plasma sTREM2 concentrations were comparable between groups. The correlation between sTREM2 levels and age, sex, Mini-Mental State Examination (MMSE) score, ASC protein concentrations, and primary CSF biomarkers for AD (total and phosphorylated tau protein and Aβ concentrations) was analyzed. CSF sTREM2 levels correlated significantly positively with patient age (r_s_ = 0.26, *p* < 0.0001; Figure 2a), plasma sTREM2 concentrations (r_s_ = 0.2, *p* = 0.02; Figure 2b), CSF p-tau_181_ (r_s_ = 0.28, *p* < 0.0001; Figure 2c), and CSF ASC protein levels (r_s_ = 0.19, *p* = 0.003; Figure 2d). CSF sTREM2 concentrations also correlated positively and highly significantly with t-tau protein levels (r_s_ = 0.31, *p* < 0.0001; Figure 2e) and negatively and significantly with MMSE score (r_s_ = −0.19, *p* = 0.002; Figure 2f). There was no relationship between the levels of sTREM2 and Aβ_1-42_ in CSF (r_s_ = 0.08, *p* = 0.18). Age was also positively correlated with plasma sTREM2 concentrations (r_s_ = 0.26, *p* = 0.002). As shown in Figure 3, the sensitivity and specificity of sTREM2 in CSF as a biomarker for AD were 65.81% and 80.00%, respectively, at a cut-off value of 23,950 pg/mL (AUC = 0.75; *p* < 0.001).

### 3.2. ASC Levels in CSF and Plasma Samples

In addition to the already mentioned positive correlation of ASC protein concentrations in the CSF with sTREM2, ASC levels were positively correlated with the age of the subjects (r_s_ = 0.22, *p* = 0.0005), p-tau_181_ concentrations (r_s_ = 0.24, *p* = 0.0003), and t-tau protein concentrations (r_s_ = 0.17, *p* = 0.01). ASC protein concentrations in plasma were positively correlated with ASC protein concentrations in the CSF (r_s_ = 0.23, *p* = 0.02) and subject age (r_s_ = 0.2, *p* = 0.02). The results of the analysis of the diagnostic potential of ASC protein showed that the concentration of ASC protein in the CSF is not a good indicator of the differences between AD patients and healthy subjects (AUC = 0.54; *p* = 0.42).

## 4. Discussion

**Figure 4:**
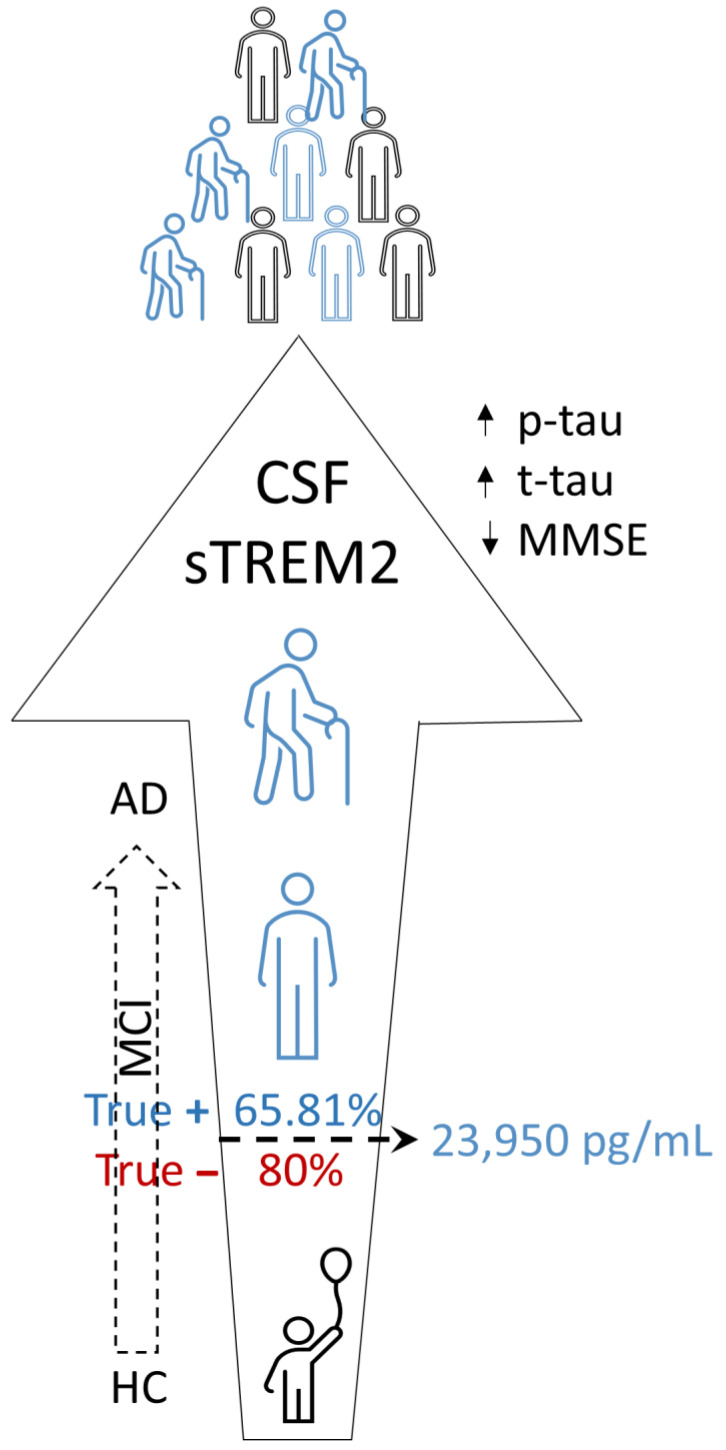
CSF sTREM2 levels increase with age and are the highest in the AD group. Higher CSF sTREM2 is associated with higher CSF levels of p-tau and total tau and lower cognitive performance. CSF sTREM2 can discriminate healthy from diseased subjects with a sensitivity of 65.81% and a specificity of 80.0%. AD, Alzheimer’s disease; CSF, cerebrospinal fluid; HC, healthy controls; MCI, mild cognitive impairment; MMSE, Mini-Mental State Examination; p-tau, tau phosphorylated at epitope 181; sTREM2, soluble Triggering Receptor Expressed on Myeloid cells 2; “true −“ denotes specificity; “true +” denotes sensitivity; t-tau, total tau proteins.

Our findings demonstrate convincingly that CSF levels of sTREM2 are significantly greater in the AD group than in the MCI and HC groups. Apart from rare exceptions [34,43], the vast majority of previous studies [44,45] have, like the present study, found elevated concentrations of sTREM2 in CSF to be strongly associated with AD. Importantly, we found that concentrations of sTREM2 in CSF are substantially higher in the MCI group compared to the HC group, confirming previous suggestions that sTREM2 could be used as a biomarker to detect conversion from MCI to AD [45] and can indicate earlier changes in the MCI stage. A conclusive graph of our main results is given in Figure 4.

Our findings also demonstrate that, statistically, plasma levels of sTREM2 do not differ between the three groups analyzed. This once again confirms that prospective AD biomarkers in plasma are poor indicators of what is occurring in the brain, and as a result, these biomarkers have unsatisfactory outcomes. There are numerous reasons for this, but some of the most important are as follows: (1) probably only a small fraction of sTREM2 enters the peripheral biofluid system; (2) like other proteins, sTREM2 can be degraded by proteases or form complexes with various blood proteins; and (3) sTREM2 can also be cleared in the liver and kidney, and by macrophages in peripheral organs and tissues [46]. The third reason suggests that the optimal blood-collection site should be changed from the cubital vein to the internal jugular vein, as this would reduce blood dilution, degradation, and organ clearance effects [46]. Regardless of the aforementioned explanations, our results indicate that plasma sTREM2 concentrations do not adequately differentiate microglial activity between the MCI, AD, and HC subject groups. On the other hand, differences in CSF sTREM2 levels suggest that the innate immune response in the brains of AD patients is much stronger and that microglia are more active in the MCI stage than during healthy aging. Using transgenic mouse models of amyloid deposition, it has been demonstrated that genetic variability in the microglial response to amyloid deposition may be a significant risk factor for AD [47]. In the same study that confirmed the involvement of four mouse orthologs of GWAS-established risk genes for AD, including *TREM2*, at least four new putative risk genes were identified with a high degree of statistical rigor, as increased expression of these genes in microglia was observed only in the presence of amyloid [47].

Previously, variations in sTREM2 levels were determined at distinct disease stages. The MCI group had the highest levels of sTREM2, which may be indicative of microglia’s response to early neuronal death [35] and efferocytosis [48]. Efferocytosis enables microglia to efficiently remove apoptotic and necrotic cells as well as cellular debris. In addition, it stimulates the production of anti-inflammatory cytokines, such as TGF-β and IL-10, while suppressing the release of pro-inflammatory cytokines. Thus, a deficiency in efferocytosis leads to the accumulation of apoptotic cells and cholesterol-rich brain tissue cellular detritus, resulting in chronic inflammation and autoimmunity [48]. Other authors [36,37] have discovered that sTREM2 concentrations are exceedingly sensitive to the onset of pathological changes during the progression of the disease [36,37,45]. In early non-symptomatic stages with low Aβ and normal tau levels, sTREM2 levels are lower, and sTREM2 concentrations increase when tau protein changes manifest as elevated CSF levels of p-tau and t-tau [36,37]. The TREM2 receptor is known to interact with Aβ and initiate its phagocytosis [11]. Ewers and colleagues [12] have demonstrated that elevated sTREM2 CSF levels prevent Aβ accumulation in the brain. In the 5xFAD model of AD, protracted TREM2 stimulation by injection of human TREM2 (hTREM2) agonistic MAb (AL002c) was protective against Aβ pathological changes and neurite damage [13]. Even though the role of the TREM2 receptor in the pathogenesis of AD is not completely understood, it is reasonable to assume that in the early stages of the disease it may play a protective role, but as the disease progresses and the adaptive immune response is more involved, it becomes harmful [11,49]. In addition, TREM2 may only be protective against a subset of the pathological alterations in AD, specifically the previously mentioned Aβ load, but not tau pathology. Recent research [49] found that the number of T cells, particularly cytotoxic T cells, was significantly elevated in regions of pathological tau changes in the brains of mice with tauopathy and AD patients.

An important study investigated 1035 participants from the Alzheimer’s Disease Neuroimaging Initiative database, including 310 HC, 527 MCI, and 198 AD subjects [50]. CSF sTREM2 levels were associated with older age and CSF p-tau and t-tau levels, all with *p* < 0.0001 [50], corroborating the findings of this investigation. Contrary to our findings, however, sTREM2 levels were not correlated with the cognitive status [50]. Another recently published study examined sTREM2 levels longitudinally from the participants of the observational Dominantly Inherited Alzheimer Network, which included families with a history of autosomal dominant AD [51]. Participants aged over 18 years were categorized as either carriers or pathogenic variants in *PSEN1*, *PSEN2*, and *APP* genes (N = 155) or non-carriers (N = 93). High amyloid burden at baseline, demonstrated by low CSF Aβ_1-42_ but not high cortical uptake in Pittsburgh compound B positron emission tomography (PiB-PET), was the only factor that indicated a higher annual rate of rise in sTREM2 in individuals with pathogenic mutations [51]. According to the authors of the study, these findings support the protective effect of sTREM2 on Aβ deposition, Aβ-dependent tau pathology, cortical atrophy, and cognitive decline [51]. As a result, sTREM2 could be an important marker for clinical trial design and interpretation.

The cross-sectional design of the present investigation is a limitation; a longitudinal study would be preferable for tracking TREM2 level changes across disease stages. Notwithstanding this, correlation analysis reveals a robust relationship between microglial activity and pathological alterations of tau proteins. In our study, sTREM2 CSF levels were positively correlated with both t-tau and p-tau_181_ CSF levels, findings in good agreement with previous studies in which tau-related neurodegeneration was reported to be associated with an increase in CSF sTREM2 (for review, see [11,27]), but not with Aβ_1-42_ CSF levels, similar to a recent report by Suarez-Cálvet et al. [36]. Based on the current investigation and the study of Suarez-Cálvet et al., it is logical to conclude that the pathology of the longest form of amyloid (Aβ_1-42_) in the absence of downstream tau-related neurodegeneration is associated with a decrease in CSF sTREM2. Intriguingly, a recent study found a positive association between sTREM2 in CSF and the shorter variants of amyloid, Aβ_x-40_ [52], which are not typically associated with AD [53,54]. These findings may be explained by the possibility that AD patients have a higher removal rate of soluble Aβ_1-42_ because of increased cellular Aβ_1-42_ uptake [55].

This interdependence between sTREM2 levels and biomarkers of tau protein alterations has been reported previously, confirming the significance of the TREM2 receptor in tauopathy pathogenesis [11]. However, the precise roles of the TREM2 receptor in tauopathies remain undetermined. Its primary functions appear to include promoting phagocytosis, moderating the response to neuronal injury, and modulating neuroinflammation [7]. Recent research examining the effect of the R47H variant on tau pathology in the PS19 mouse model of tauopathy revealed that this variant improves microglia response and causes neurodegenerative alterations related to pathological changes of tau proteins [30]. In the THY-Tau22 mouse model of tauopathy, another study revealed that TREM2 deficiency worsens tau pathological changes in later stages owing to the lower microglial activation rate [32]. Depending on the *APOE* genotype, TREM2 signaling has distinct effects on pathological changes in the tau protein. Tau pathological changes and neurodegeneration are significantly more severe in mice with a silenced *TREM2* gene and ApoE4 than in mice with an active *TREM2* gene [31]. This is further evidence of the complexity of TREM2 signaling and a reminder of the distinctions between animal models of AD and humans. A positive correlation between sTREM2 concentrations and the age of the subjects suggests that microglial activation increases with age, whereas a negative correlation between sTREM2 concentrations and the MMSE score suggests that sTREM2 could be a good indicator of cognitive deficits and that neurodegenerative changes and microglial activation are highly interrelated.

In addition, sTREM2 CSF levels were positively correlated with CSF ASC protein levels, indicating a potential link between the release of the soluble TREM2 form and the activation of the inflammasomes. Multiple studies [19,20,21,23] have demonstrated the relationship between inflammasomes and TREM2 receptors. Microglia cells with the R47H *TREM2* gene variant, which increases the risk of AD onset, have a decreased activation of the NOD-like leucine-rich repeat receptors family pyrin domain-containing 3 (NLRP3) inflammasome upon ligand binding to the TREM2 receptor [19]. Overexpression of *TREM2* in macrophages inhibits activation of the NLRP3 inflammasome [20], whereas silencing the *TREM2* gene substantially increases macrophage pyroptosis [21]. These results suggest that TREM2 receptor expression performs a protective and anti-inflammatory role (for review, see [56]). In the context of AD, this property of TREM2 implies greater control over the inflammatory response and protection from over-activation and cross-activation of the inflammasomes, including the neuronal NLRP1 inflammasome [57], but this does not imply that silencing the inflammatory response is always the best course of action. For a healthy immune response, the timing and sequence of events are of the utmost importance, as AD pathological changes may affect the interaction between two receptors differently. In interpreting the results, it is important to remember that a previous study [58] has already demonstrated a correlation between elevated sTREM2 levels and pathological changes of the white matter, specifically small vessel disease and amyloid angiopathy, regardless of the severity of other pathological changes. This study demonstrates that CSF sTREM2 concentrations can differentiate between healthy and AD patients. With a cutoff value of 23,950 pg/mL, CSF sTREM2 had a relatively good sensitivity of 66.81% and a high specificity of 80%.

Excessive inflammasome activation is one of the adverse effects of chronically active microglia. The AD group had higher concentrations of ASC protein in the CSF than the MCI and HC groups, but the differences in plasma concentrations were not statistically significant. In addition, there was no significant difference between the MCI and HC groups in terms of CSF ASC protein concentrations. This indicates that inflammasome activity is greater in AD compared to MCI, where significant activation of the inflammasome and release of ASC protein into the extracellular milieu does not likely occur. Even though the concentrations of ASC in the CSF are significantly higher in the AD group, the diagnostic potential analysis of the ASC protein revealed that the concentration of the ASC protein in the CSF is not a reliable diagnostic marker for distinguishing AD patients from cognitively healthy subjects. In addition to microglia, we know that neurons also express inflammasomes. In neurons, the NLRP1 inflammasome is especially active [55], so higher concentrations of extracellular ASC in AD may be a result of more severe neuronal degeneration. In contrast to the findings of the present research, a recent study found elevated concentrations of ASC protein in the serum of MCI subjects compared to those of control and AD subjects [59]. The extended period of storage of plasma samples in our study, as well as the small number of subjects in the HC group, could account for conflicting results. Moreover, the presence of ASC protein in peripheral blood is not only an indicator of inflammasome activation in the brain but also of inflammatory processes in peripheral tissues. In addition to the previously mentioned concentrations of sTREM2 in the CSF, the concentrations of ASC protein in the CSF were positively correlated with the concentrations of ASC in the plasma and the age of the subjects, which can be interpreted as the activation of the inflammasome becoming more pronounced with age. Similar to sTREM2, the concentrations of ASC protein in the CSF of our sample are positively correlated with t-tau and p-tau_181_ concentrations, confirming the interdependence between the activation of inflammatory processes and pathological alterations of tau protein. It is known that microglia in AD are activated by the presence of pathologically altered tau protein and that pathological forms of tau protein in neurodegenerative diseases influence the course of inflammation [60,61]. Additionally, it has been shown that aggregated forms of tau protein activate the NLRP3 inflammasome [62]. As a result of the phagocytosis of aggregated forms of tau protein and the inability of microglia to degrade them, the inflammasome is activated, and it has been proposed that microglia also contribute to the spread of neurofibrillary changes [62]. The aforementioned findings indicate that the inflammasome can be activated by the consumption of aggregated ASC and altered tau proteins in nearby cells [62]. This results in the dissemination of inflammation and pathological tau protein changes via tau seeds, which serve as tau templates for the pathological folding of normal tau monomers throughout the affected regions of the brain [56]. Although the precise sequence and cause-and-effect relationships of pathological changes in the activation of the inflammasome and tau protein have not been fully elucidated, the aforementioned findings confirm the importance of further research in this field.

AD is also characterized by dysregulation of glucose metabolism [63,64,65,66], and a recent study found that inflammation initiated by elevated glucose levels in the BV2 microglial cell line results in a higher rate of NLRP3 inflammasome activation [23]. Additional experiments have shown that the TREM2 receptor can modulate the inflammatory response initiated by high glucose levels and the NLRP3 inflammasome [23]. Choi and colleagues found that 5xFAD mouse microglia consume significantly more glucose than wild-type microglia and that as the disease progresses, microglia express a greater number of glucose transporters (GLUT1 and GLUT2), have insufficient glycolysis, and have a higher level of oxidative phosphorylation [28]. In addition, they discovered a positive correlation between the levels of CSF sTREM2 and glucose intake in the human hippocampus [28], which, given the activation of inflammasomes in glia in response to high glucose concentrations, could be a link between sTREM2 levels and the activation of inflammasomes. Despite the apparent connection between TREM2 signaling and the inflammasome activation pathway, the role and interaction of sTREM2 and inflammasome activation remain undefined. Even though other monocytes can also express the TREM2 receptor and release its soluble form into the blood [67], our results indicate a positive correlation between CSF and plasma sTREM2, which suggests that the state of the periphery indicates alterations in the brain to some extent. Several studies have failed to find a correlation between CSF sTREM2 levels and peripheral blood sTREM2 levels [68,69], and one study reported a negative correlation between CSF and plasma sTREM2 levels [70]. Despite obvious differences in CSF sTREM2 levels, the latter statement can explain the lack of significant differences in plasma sTREM2 levels between the analyzed groups due to the reasons already discussed (for review, see [46]). Lastly, plasma sample groups were substantially smaller than CSF sample groups, which may explain why the differences were not statistically significant. The major limitation of the study is the small number of samples in some groups, especially plasma samples. Additionally, plasma samples were stored for a longer period than CSF samples before the analysis of sTREM2 and ASC protein levels.

## 5. Conclusions

In contrast to plasma sTREM2, CSF sTREM2 levels were significantly higher in the AD group than in the MCI and HC groups, indicating a high diagnostic potential for distinguishing diseased from healthy individuals. The significant difference in sTREM2 levels between patients with MCI and HC subjects highlights sTREM2’s potential even further. AD patients with elevated CSF sTREM2 levels reliably predict neurofibrillary degeneration, cognitive decline, and inflammasome activation. To confirm the addition of CSF sTREM2 to the list of mandatory biomarkers for AD, future research should include more patients and follow the same pre-analytical, analytical, and post-analytical procedures. Considering the complexity of TREM2 signaling in relation to various pathological aspects of AD, future longitudinal studies on AD patients are necessary.

## Figures and Tables

**Table 1:** Age and number of Mini-Mental State Examination (MMSE) points, and cerebrospinal fluid (CSF) levels of sTREM2, t-tau, p-tau_181_, Aβ_1-42_, and ASC for the AD, MCI, and HC groups, where (n) represents the sample size analyzed.

Group	Age (Years) Mean ± SD (n)	MMSE (Points) Mean ± SD (n)	sTREM2 (pg/mL) Mean ± SD (n)	ASC (ng/mL) Mean ± SD (n)	Aβ_1-42_ (pg/mL) Mean ± SD (n)	p-tau_181_ (pg/mL) Mean ± SD (n)	t-tau (pg/mL) Mean ± SD (n)
AD	70.58 ± 8.670 (154) ^a,b^	20.30 ± 4.395 (142) ^a,b^	33,854 ± 19,631 (155) ^a,b^	3.817± 1.348 (126)	549.5 ± 303.9 (152)	77.06 ± 45.25 (147) ^a,b^	522.5 ± 400.9 (146) ^a,b^
MCI	66.42 ± 8.832 (90) ^a,c^	25.64 ± 2.903 (74) ^a,c^	26,940 ± 17,043 (90) ^a,c^	3.432 ± 1.121 (78)	665.9 ± 374.8 (86) ^c^	53.32 ± 29.90 (87) ^a,c^	257.5 ± 177.1 (86) ^a^
HC	58.80 ± 10.68 (49) ^b,c^	29.06 ± 2.199 (32) ^b,c^	18,527 ± 13,566 (50) ^b,c^	3.369 ± 1.272 (39)	552.8 ± 521.7 (49) ^c^	35.18 ± 22.70 (49) ^b,c^	222.7 ± 284.0 (48) ^b^
Kruskal–Wallis test	H test = 45.13 *p* < 0.0001	H test = 124.4 *p* < 0.0001	H test = 35.38 *p* < 0.0001	H test = 6.44 *p* = 0.04	H test = 8.873 *p* = 0.01	H test = 57.40 *p* < 0.0001	H test = 58.02 *p* < 0.0001

^a^ Significant difference between AD and MCI; ^b^ Significant difference between AD and HC; ^c^ Significant difference between MCI and HC.

## Data Availability

All the data reported are available on request from the corresponding author.
